# Inhaled nano-based therapeutics for pulmonary fibrosis: recent advances and future prospects

**DOI:** 10.1186/s12951-023-01971-7

**Published:** 2023-07-08

**Authors:** Qianyu Wan, Xinrui Zhang, Dongfang Zhou, Rui Xie, Yue Cai, Kehao Zhang, Xuanrong Sun

**Affiliations:** 1 Collaborative Innovation Center of Yangtze River Delta Region Green Pharmaceuticals and College of Pharmaceutical Science, Zhejiang University of Technology, Hangzhou, 310014 China; 2 Zhejiang China Resources Sanjiu Zhongyi Pharmaceutical Co., Ltd, Lishui, 323000 China

**Keywords:** Inhalation, Nano-based drug delivery system, Pulmonary fibrosis, COVID-19

## Abstract

It is reported that pulmonary fibrosis has become one of the major long-term complications of COVID-19, even in asymptomatic individuals. Currently, despite the best efforts of the global medical community, there are no treatments for COVID-induced pulmonary fibrosis. Recently, inhalable nanocarriers have received more attention due to their ability to improve the solubility of insoluble drugs, penetrate biological barriers of the lungs and target fibrotic tissues in the lungs. The inhalation route has many advantages as a non-invasive method of administration and the local delivery of anti-fibrosis agents to fibrotic tissues like direct to the lesion from the respiratory system, high delivery efficiency, low systemic toxicity, low therapeutic dose and more stable dosage forms. In addition, the lung has low biometabolic enzyme activity and no hepatic first-pass effect, so the drug is rapidly absorbed after pulmonary administration, which can significantly improve the bioavailability of the drug. This paper summary the pathogenesis and current treatment of pulmonary fibrosis and reviews various inhalable systems for drug delivery in the treatment of pulmonary fibrosis, including lipid-based nanocarriers, nanovesicles, polymeric nanocarriers, protein nanocarriers, nanosuspensions, nanoparticles, gold nanoparticles and hydrogel, which provides a theoretical basis for finding new strategies for the treatment of pulmonary fibrosis and clinical rational drug use.

## Introduction

### Pulmonary fibrosis

**Fig. 1:**
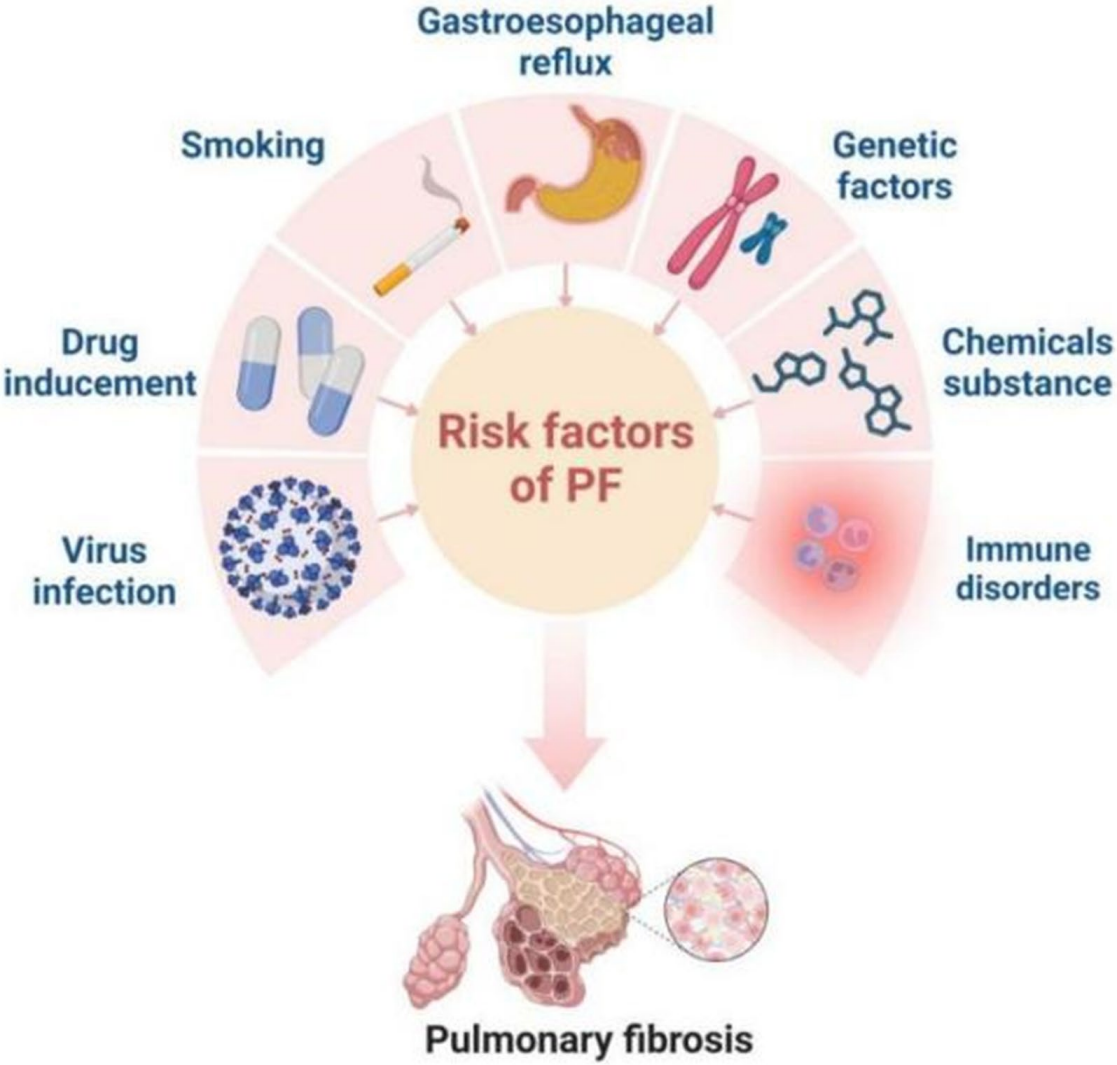
The relatively clear etiology and predisposing factors leading to PF

Pulmonary fibrosis (PF) is a common, progressive, irreversible, fatal chronic pulmonary disease [1, 2] with a median survival of 2–4 years after diagnosis [3], which is characterized by excessive extracellular matrix deposition and scar in the lungs, resulting in functional failures, severe breathing problems and even death [4, 5]. It can be divided into secondary PF and idiopathic PF [6]. The relatively clear etiology and predisposing factors for PF are as follows: smoking, gastroesophageal reflux, genetic factors, some chemicals (e.g. organic or inorganic dust), some drugs (e.g. amiodarone and bleomycin), viral infections and some immune disorders (e.g. lupus erythematosus and scleroderma) [7] (Fig. 1).

**Fig. 2:**
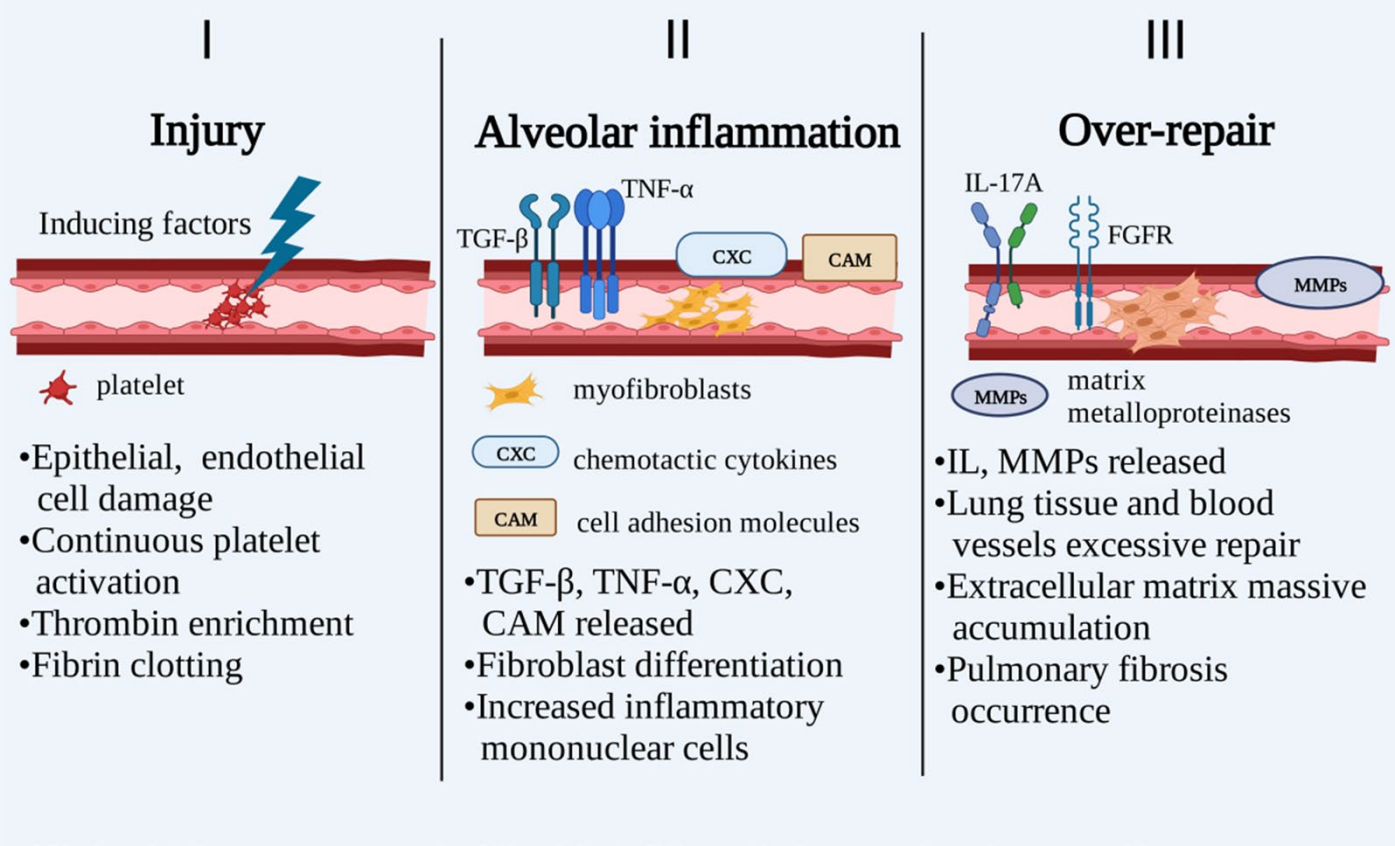
Schematic diagram of PF process and development, including three main stages: injury, alveolar inflammation, fibrosis and excessive repair

Studies have concluded that the pathogenesis of PF is divided into three stages [7, 8]: (1) Injury: many factors can induce lung injuries, such as smoking, gastroesophageal reflux, genetic factors, some chemicals, some drugs, and viral infections. The destruction of epithelial and endothelial cells triggers the anti-fibrinolytic cascade reaction. Platelets are continuously activated, thrombin is enriched, and fibrin clots. (2) Alveolar inflammation: lung activated mesenchymal and infiltrating cells secrete transforming growth factor-β (TGF-β), tumor necrosis factor-α (TNF-α), chemotactic cytokines (CXC), and cell adhesion molecule (CAM). These cytokines promote an increase in inflammatory monoclonal factors. (3) Fibrosis with the excessive repair: fibroblast and myofibroblast activation, secretion of growth factors, interleukins (IL)-17A, and matrix metalloproteinases (MMPs), fibroblast growth factor receptor (FGFR), etc. Fibrosis occurs with the excessive repair of lung tissue and blood vessels, excessive proliferation of fibroblasts, and massive accumulation of extracellular matrix (Fig. 2). In late December 2019, COVID-19 outbroke and spread to several countries, resulting in a cumulative total of over 400 million confirmed cases, with the complications and sequelae of PF in critical and severe illnesses [8–11].

### Treatments currently available of PF

However, there is a lack of effective clinical treatment to completely cure PF. Symptomatic treatment, such as the use of glucocorticoid and anti-infection therapy, is generally used. The prognosis of PF is unfavorable, which seriously threatens the life and health of human beings. In 2014, two anti-fibrotic drugs, pirfenidone and nintedanib, have been approved for use in humans by FDA [12], these drugs only can slow the decline of lung function but do not cure or reverse established fibrosis [13]. As a result, seeking a proper treatment for PF leads to a great deal of attention.

**Fig. 3:**
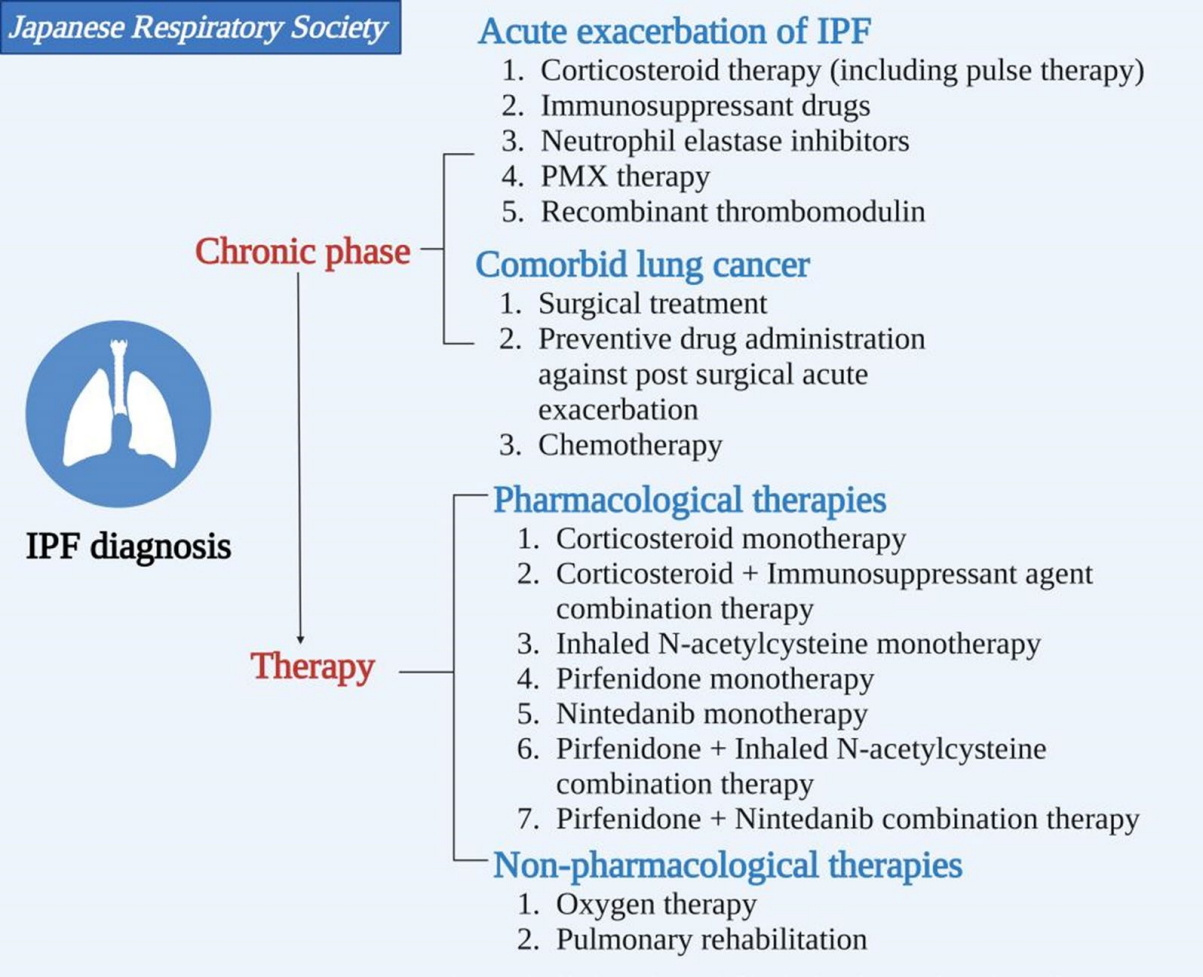
The latest international treatment guideline was presented by the JRS in 2018

The latest international treatment guideline was presented by the Japanese Respiratory Society (JRS) in 2018, giving a conditional recommendation in PF treatments, containing pirfenidone combined with inhaled N-acetylcysteine therapy, inhaled *N*-acetylcysteine therapy, corticosteroid monotherapy, etc. (Fig. 3) [14]

### Inhaled nano-based therapeutics for PF

Notably, the long-term dosing and systemic administration of these drugs can cause serious side effects, such as diarrhea [15], nausea [16], vomiting [17] and rash/photosensitivity [18]. However, nano-based drug delivery systems can improve the solubility and bioavailability of anti-fibrotic drugs and immunotherapeutic agents, increase drug uptake by target cells, increase lung deposition rates, prolong the retention time in vivo and improve pharmacokinetic behavior, thereby significantly enhancing the therapeutic effect at low doses of anti-fibrotic drugs and reducing side effects [19, 20]. Inhalation nano-based drug delivery system as an emerging therapy offers a new way of treating PF, compared with traditional oral administration, inhalation administration can make the drug diffuse throughout the lung and promote bronchial relaxation. This kind of administration can greatly reduce the drug dosage, and the drug can reach the lung without passing through the blood circulation, with a high deposition rate in the lung.

**Fig. 4:**
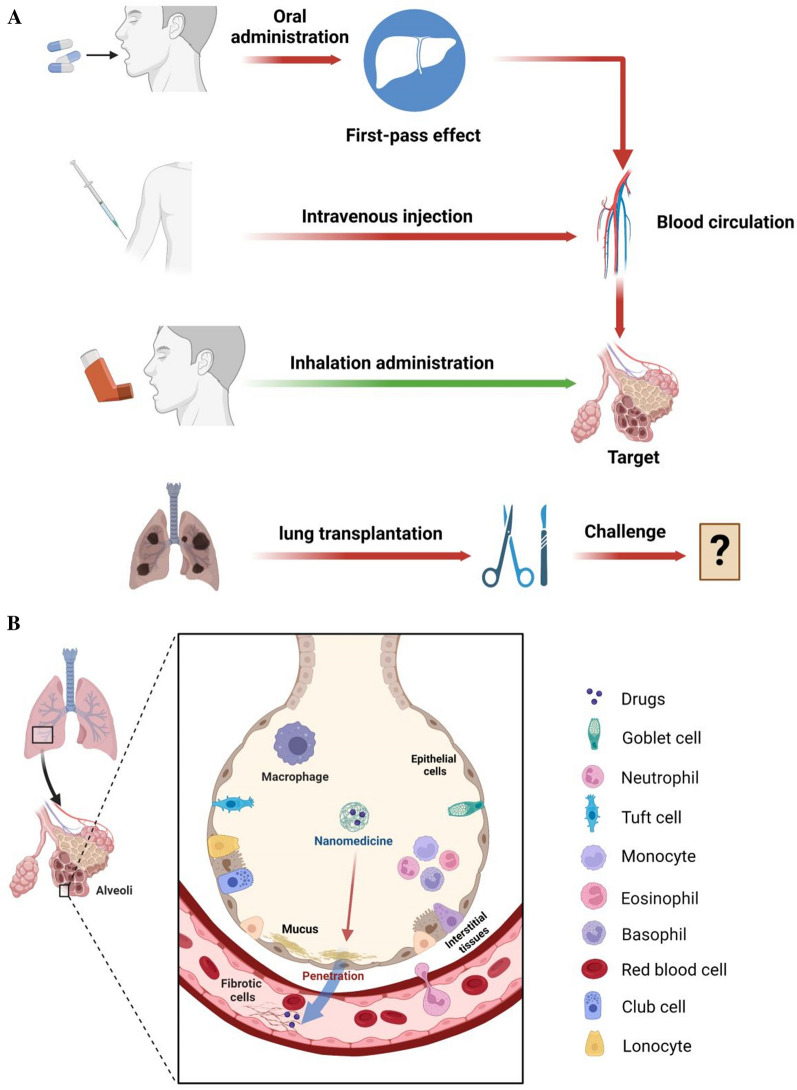
**A** Series routes for the treatment of PF, including inhalation, intravenous, oral administration and lung transplantation. **B** Several biological barriers that need to be penetrated for inhaled nanomedicines to treat PF: the nanomedicines are inhaled and diffused into the alveoli, and then sequentially cross mucus, alveolar epithelial cells, interstitial tissues, capillary endothelial cells, plasma, and finally, reach the capillary red blood cells

Inhalation also has several meaningful advantages over injections, such as improved patient compliance, low therapeutic dose, safety, and treatment outcomes. Nano-based drug delivery system can wrap anti-fibrosis drugs in biocompatible materials. The nanomaterial shell has a protective effect, but also can effectively improve the stability of active ingredients and drugs. It has good biocompatibility and can improve bioavailability. After inhalation, the nanodrug first penetrates the lung barrier, and then accumulates to the target after endocytosis, and then releases the drug, and finally enters the blood circulation [21, 22]. This emerging delivery system can reduce the number of doses to achieve a sustained release therapeutic effect (Fig. 4A, Table 1).

**Table 1:** Comparison of present treatment methods for PF

Administration route	Advantages	Disadvantages
Intravenous injection	Time-saving, larger dosage [32], directly into the blood circulation, avoid first-pass effect; Can be used on unconscious patients	Inconvenience; Strong treatment compliance, risk of infection; Trained medical staff needed
Oral administration	Common, convenient, relatively cheap	Slow absorption, not suitable for first aid, lesser extent affect liver functions [33], low lung deposition; More adverse effects
Lung transplantation	The only intervention that improves survival in advanced PF	Existing chronic rejection reaction, risk of postoperative infection, more comorbidities, uncertain transplantation waiting time [34]
Conventional inhalation administration	Fast effect speed, avoid the first-pass metabolism, controlled dose	Irritate the lung epithelium, most free drugs, natural nucleic acids and peptides cannot enter the lungs through inhalation [35]
Nano-based inhalation administration	A enhancement of the treatment efficiency, limiting possible adverse effects upon other healthy organs [35], with good targeting, biological activity and penetration ability	Facing the challenge of nanotoxicity, such as local inflammation and cytokine storm [36]. High requirements for particle morphology, size and charge

On inhalation, the nanomedicines require the penetration of several physiological barriers: first inhaled and diffused into the alveoli, and then penetrate the mucus, alveolar epithelial cells, interstitial tissues, capillary endothelial cells, and plasma, and finally, reach the capillary red blood cells (Fig. 4B) [23, 24]. Unfortunately, little is known about the interaction of inhalation drug delivery with biological membrane, especially with extracellular polymeric substances (EPS) matrices [25], so there are still some safety issues with nano formulation, such as causing local inflammation and cytokine storms.

**Fig. 5:**
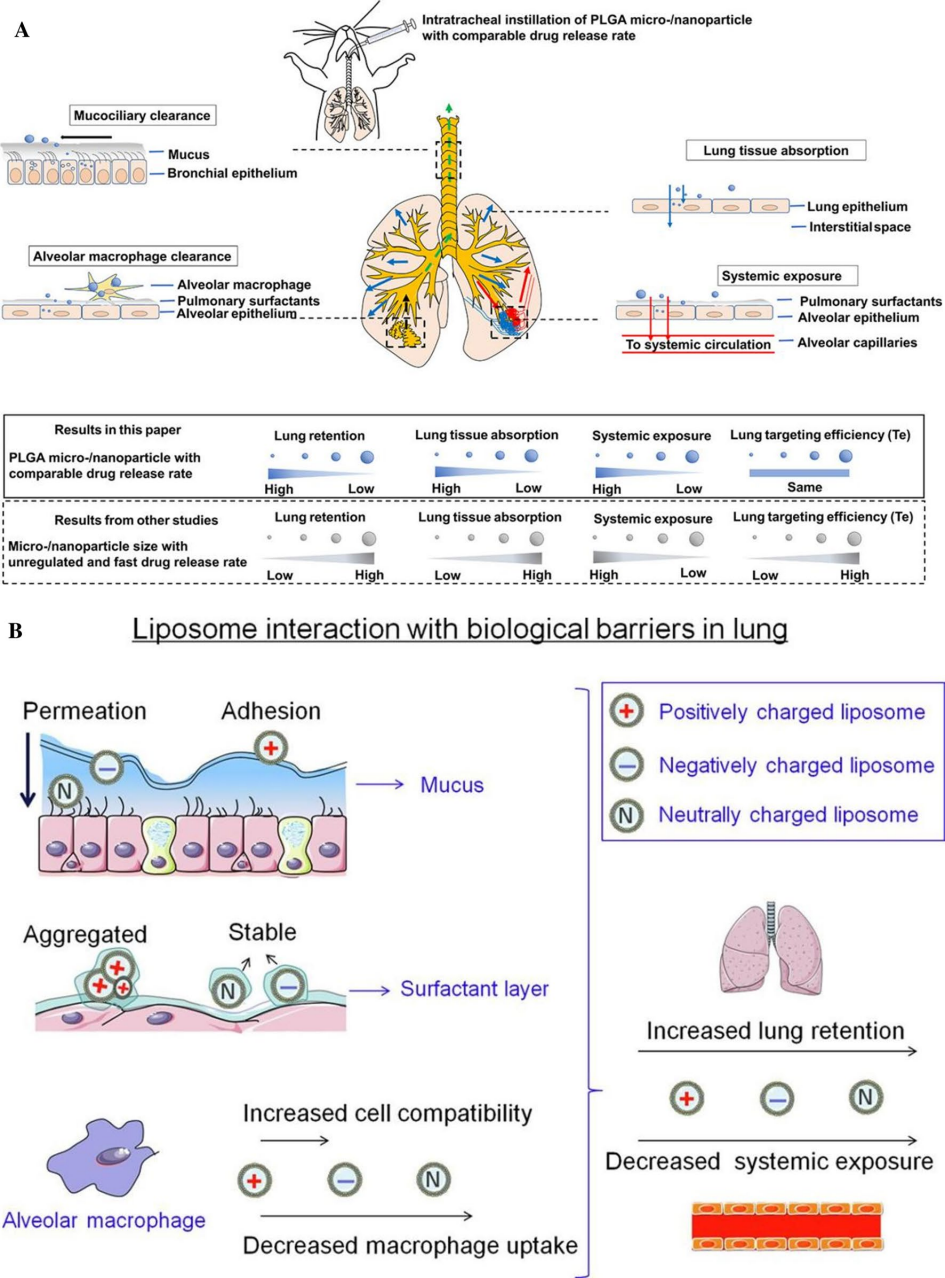
**A** Comparison of optimal particle sizes for pulmonary inhalation drug delivery, e.g. lung retention time, lung tissue absorption, systemic exposure and pulmonary targeting efficiency, etc. [26]; **B** Charge selection for pulmonary inhalation drug delivery, e.g. cytocompatibility, lung retention time, systemic exposure and mucus penetration, etc. [28]

The challenges of inhalation drug delivery also has high demands on factors such as exposure time, dose, aggregation, concentration, particle size, surface area and charge are all closely related to nanotoxicity. The optimum particle size for inhalation is 100–500 nm (Fig. 5A), at around 100 nm, nanoparticles are more readily taken up by lung epithelial cells [26] and can be deposited in deep lung areas, particularly in alveolar structures near alveolar openings [27], if the mass median aerodynamic diameter (MMAD) of particles is less than or equal to approximately 500 nm, they are significantly deposited by diffusion based on Brownian motion [27], and if they exceed 500 nm, they are readily phagocytosed by macrophages and tend to be deposited in the alveolar lumen [27]. In addition, surface electrostatic charge is an important factor influencing the deposition of inhaled nanoparticles. Charged nanoparticles have a higher deposition efficiency compared to neutral charged nanoparticles [27].

Zhao et al. [28] reported that negatively charged and neutral nanoparticles showed better lung cytocompatibility, higher cell viability and higher mucus permeability compared to positively charged nanoparticles (Fig. 5B). In contrast, cationic drug delivery systems typically showed cytotoxicity, which limited the dose administered [29].

It should not be ignored that inhaled nano-based drug delivery system has certain limitations, which requires high parameters such as particle shape, size and charge, so it has certain challenges in design. At the same time, changes in the physicochemical and structural properties of nanocarriers may be responsible for many material interactions, which may lead to toxicological effects [30], i.e. nanotoxicity, such as causing unwanted oxidative stress and cytotoxicity [31]. Efficient targeting of lung fibrotic cells and improving efficacy are key issues that need to be addressed by the new generation of inhaled nanoformulations. The above problems are the limitations of inhaled nanoformulations and need further research and improvement.

**Fig. 6:**
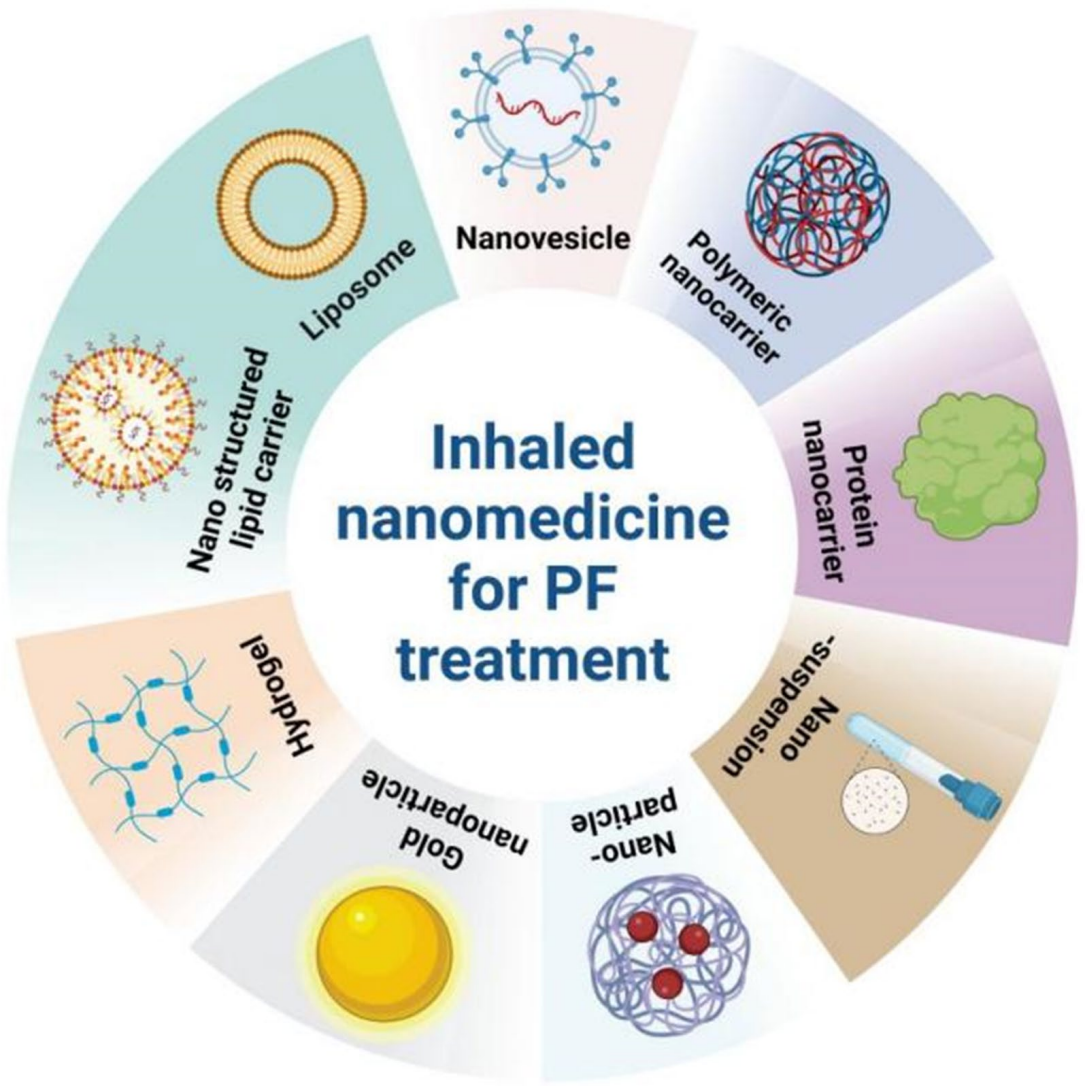
The classification of inhaled nanomedicine for PF treatment, including nanostructured lipid carriers, nanovesicles, polymeric nanocarriers, protein nanocarriers, nanosuspensions, nanoparticles, gold nanoparticles and hydrogel

The review here presents the current application of inhaled nano-based therapeutics such as lipid-based nanocarriers, nanovesicles, polymeric nanocarriers, protein nanocarriers, nanosuspensions, nanoparticles, gold nanoparticles and hydrogel in the treatment and management of PF and future prospects of this emerging field (Fig. 6).

## Lipid-based delivery system

Liposomes are one of the most successful inhalable nanocarriers among lipid matrix carriers. The hydrophilic drugs can be concentrated in the water core, while the hydrophobic drugs are mainly distributed in the lipid bilayer. Liposomes can improve intracellular uptake, prolong drug release, and reduce lung clearance rate. In addition, due to the biocompatibility and biodegradability of liposomes, there are nearly little local irritation and toxicological problems that occurs [37].

### Liposomes

Liposomes are typically made from compounds that are structurally similar to those found in the lung, such as cholesterol and phospholipids [38]. This colloidal form includes self-assembled microvesicles consisting of an aqueous core surrounded by one or more lipid bilayers. Many studies have determined the high biodegradability and biocompatibility of liposome agents for drug delivery in the lungs [39]. Ivanova et al. [40] confirmed that liposome lung local delivery of prostaglandin E2 (PGE2), which prevents impaired expression of many genes associated with the development of IPF, significantly reduces inflammatory and fibrotic lung tissue damage, reduces hydroxyproline (HYP) accumulation in the lung, and virtually eliminates mortality in animals after tracheal bleomycin infusion. Lammers et al. [41] aid to increase anti-COVID-19 PF complications therapeutic efficiency by nano-formulating dexamethasone and giving it by inhalation by targeting the interleukin receptor, potentiating its anti-oedema activity and by utilizing its anti-fibrotic effects. It is striking that it may contribute to better control of the severity of PF and promote better management of life-threatening symptoms in the acute and intermediate stages.

**Fig. 7:**
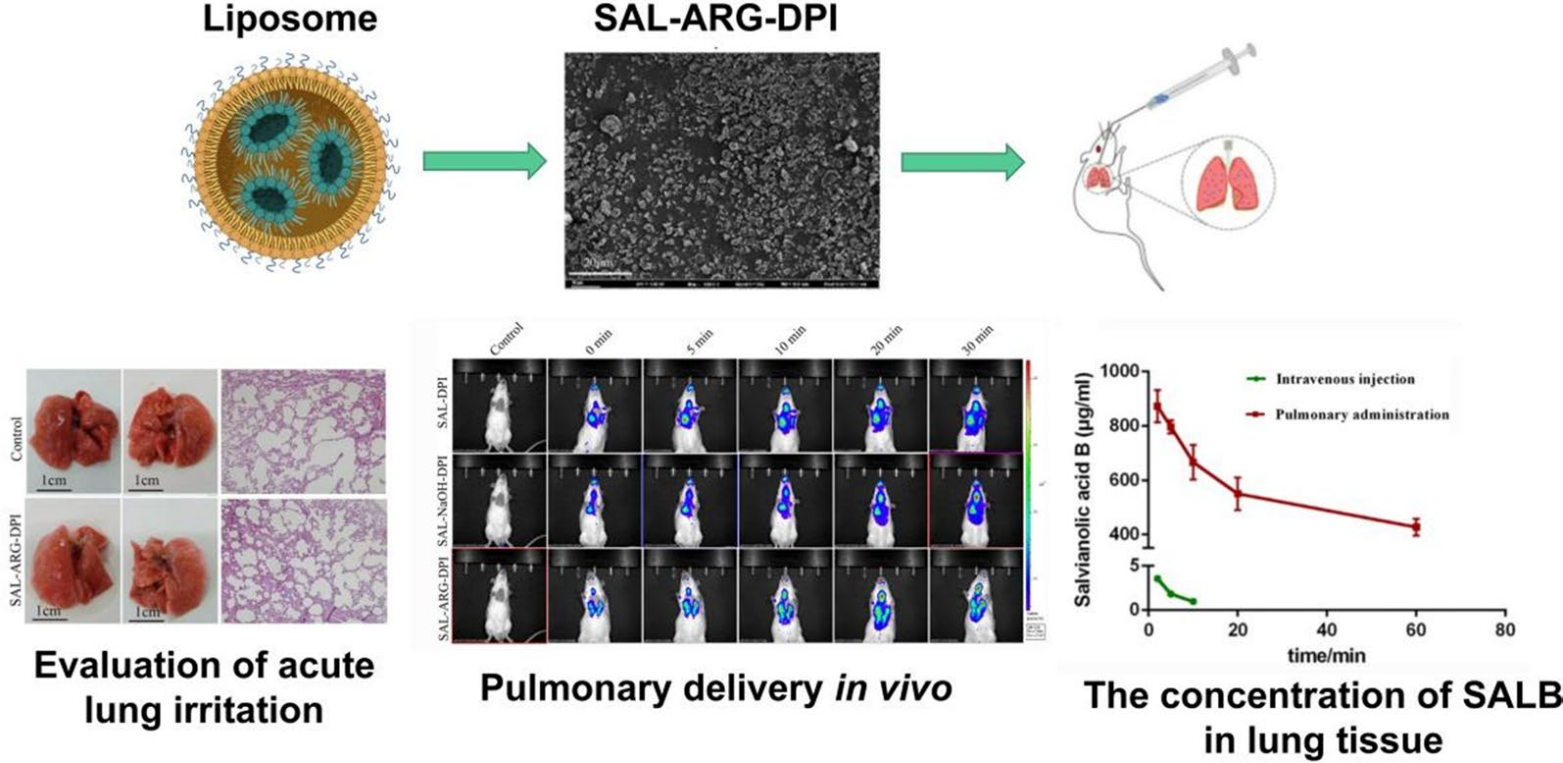
Schematic of salvianolic acid dry powder inhaler for the treatment of PF. Salvianolic acid, arginine and lecithin dry powder were prepared by ball milling technology and successfully delivered to the lungs after aerosolization. The study proved that the biocompatibility, bioavailability and lung deposition rate were improved [43]

In studies for the treatment of PF, natural medicines such as herbs have shown low adverse effects, stable anti-inflammatory and anti-fibrotic therapeutic effects and no significant drug dependence due to their natural properties. This makes them a promising therapeutic target for PF [42]. Jiang et al. [43] took advantages of the ball milling technique to prepare a salvianolic acids dry powder inhalation complex with l-arginine and lecithin. It brings out good flowing properties, excellent biocompatibility, high deposition in the lung. The lung tissue Area under the concentration–time curve between 0 and 1 h (AUC_(0–1)_) of the pharmacokinetic concentration—time curve was 2099.12 times higher than that of the intravenous administration group (Fig. 7). Chennakesavulu et al. [44] have reported liposomal dry powder inhalants of budesonide and colchicine complex. The encapsulation rate of lyophilized liposomes using mannitol as the carrier was 97.89–98.6%. The system also supplied prolonged drug retention (more than 24 h) at targeted sites and reduced systemic exposure. Kotta et al. [45] employed an endogenous surfactant-based liposome delivery system, encapsulating Naringin, which acts as an aerosol to support lung mechanics in the management of PF, showing a 79 ± 1.5% lung deposition rate as well as low toxicity. Zhou et al. [46] designed a nebulized paclitaxel liposome inhalation system for the treatment of bleomycin-induced PF in rats. The results show that inhaled paclitaxel liposomes can prevent severe PF. This method of administration has fewer systemic side effects and is safer than intravenous injection.

**Fig. 8:**
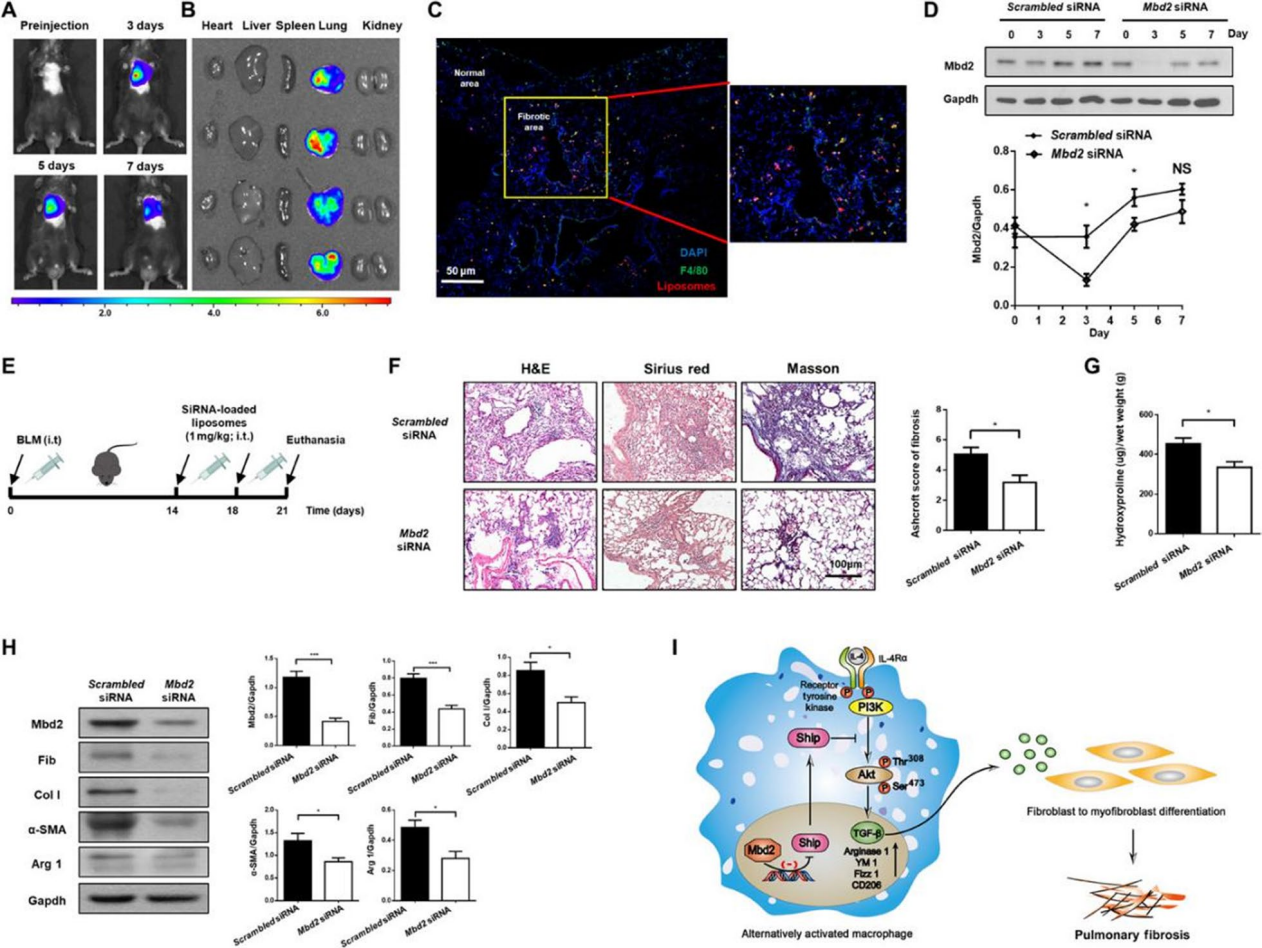
Intratracheal administration of MBD2 siRNA-loaded liposomes protects mice from BLM-induced lung injury and fibrosis [48]. **A** IVIS images of the mouse after inhaled liposomes; **B** The distribution of liposomes in the body organs; **C** Confocal immunofluorescence image of liposome in mice with PF; **D** Temporal MBD2 expression changes in the lungs from liposome-administered mice; **E** Schematic for experimental design and administration time line; **F** Intratracheal administration of liposomes loaded with MBD2 siRNA protected mice from lung injury and fibrosis. Left: Representative results of staining. Right: Ashcroft scores associated with fibrosis severity. **G** Determination of HYP in mice after PF. **H** Western blot analysis of the expressions of MBD2, fibronectin, type I collagen, α-SMA and arginase 1 in lung tissue. **I** MBD2 selectively binds Ship promoter in macrophages, enhances PI3K/Akt signaling pathway and promotes macrophage M2 program by inhibiting Ship expression

Emerging targets represent a new breakthrough for the treatment of PF and can potentially be encapsulated in advanced drug delivery formulations for site-specific therapy [17]. Pandolfi et al. [47] modified liposome with hyaluronic acid, which plays a vital role in its specific receptor CD44, overexpressed in pulmonary fibrotic cells. The liposomes decreased pro-inflammatory cytokines IL-1β, IL-12 and anti-fibrotic VEGF transcripts, but increased TGF-β mRNA. Wang et al. [48] intratracheal administrated methyl-CpG-binding domain 2 (MBD2) siRNA liposomes to protect mice against bleomycin-induced PF. The entrapment efficiency of prepared liposomes displayed approximately 90% for loading siRNA. The toxicity of liposome-loaded siRNA in cell viability was not detected. Those liposomes that were administered intratracheal continued to accumulate in the lungs for at least 7 days. NIRF signals were only detected in the lungs but absent in other organs (Fig. 8). Polyethylene glycol (PEG)ylated liposomes may also be considered in formulations characterised by reduced renal filtration, reduced uptake by the reticuloendothelial system (RES, e.g. liver and spleen) and reduced enzymatic degradation. As a result, PEGylated drugs show an extended half-life in vivo, resulting in enhanced bioavailability [49].

Togami et al. [50] used a aerosolized drug delivery system with PEGylated liposomes to improve the pharmacokinetic properties of the drug, which could effectively prolong the distribution of the drug in the lungs and could be an effective delivery system for antifibrotic drugs.

### Nano structured lipid carrier (NLC)

**Fig. 9:**
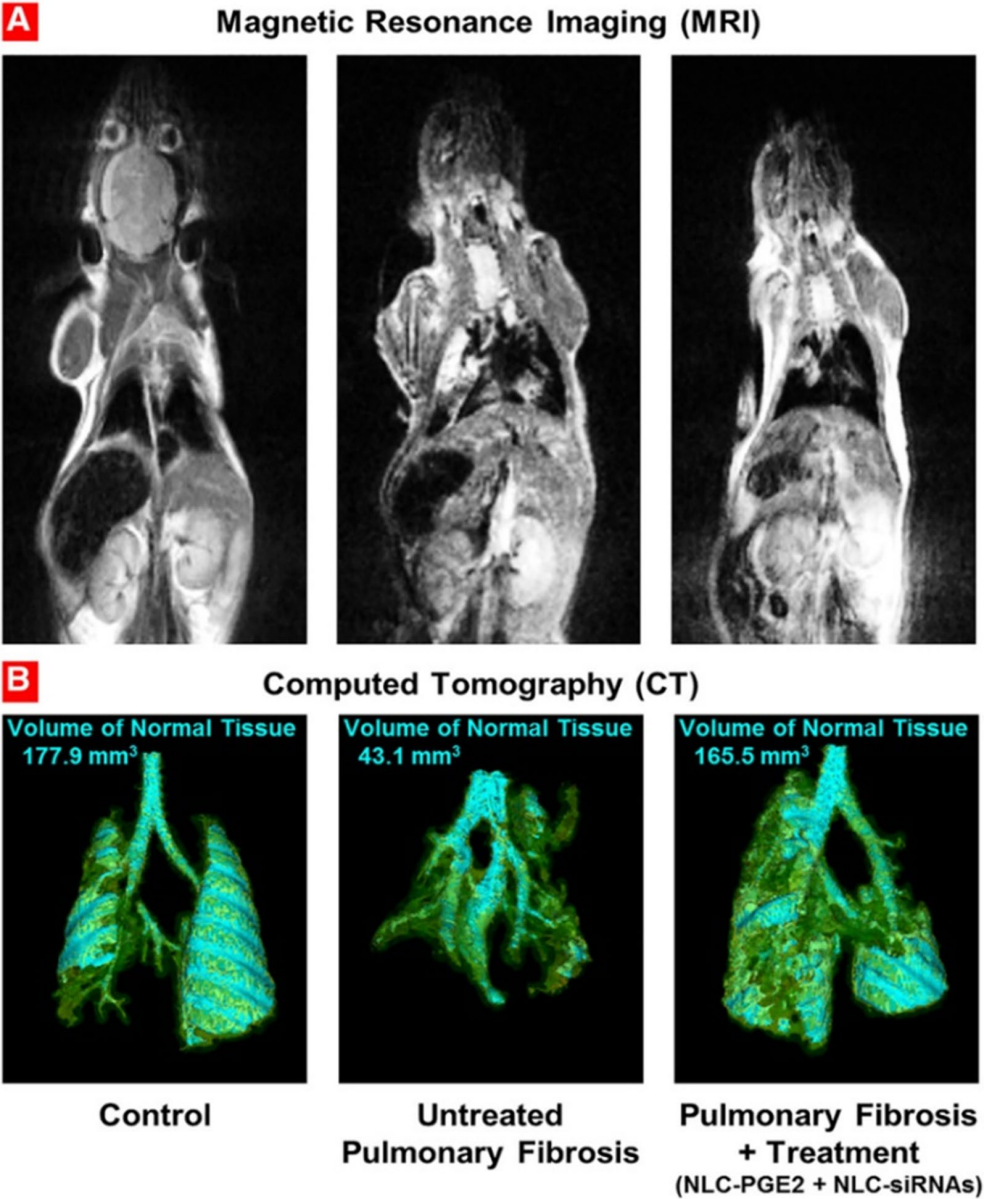
MRI and CT comparison of the development of PF in the healthy and untreated groups and in the PGE2 and siRNA nanostructured lipid carrier treatment groups. The areas with cyan color represent normal lung tissue. Yellow indicates normal connective tissue, while green indicates fibrotic tissue. After treatment, the volume of fibrotic tissue in the lungs was reduced by 3.8 times, which is almost the same as the normal value [52]

The NLC is made up of an aqueous phase with a surfactant and an unstructured solid lipid matrix (a combination of solid and liquid lipids). Because NLC uses lipid mixtures, it is not easy to form an orderly crystal structure, which can give more sealing space to the drug. NLC addresses the problems of low solid lipid nanoparticles (SLN) drug load and easy drug leakage while having the slow-release advantage of SLN [51]. For the effective treatment of idiopathic PF, Garbuzenko et al. [52] presented a prostaglandin E (PGE2) administered in combination with selected siRNAs (CCL12, MMP3, and HIF1A) by inhalation using Precirol ATO 5, which is a solid lipid as carrier. After treatment, the volume of fibrotic tissue in the lungs was reduced by 3.8-fold, which was essentially the same as the normal value (Fig. 9). The combination treatment also suppressed the expression of all pro-fibrotic genes, such as the expression of CTGF, TGFB1, TGFB2, TGFB3, TGFBR1, and TGFBR2 was downregulated. Keum et al. [53] evaluated the therapeutic efficacy of APTATstat3-9R, a high-affinity peptide conjugate that inhibits signal transducer and activator of transcription 3 (STAT3) phosphorylation via cell-penetrating 9-arginine motif modifications, a STAT3 phosphorylated cell-permeable peptide inhibitor, for the indication of improving PF. The biomimetic lipid nanocomplexes inhibits M2 polarization of lung epithelial and fibroblast differentiation into myofibroblasts and macrophages and penetrates the lung surfactant barrier and is taken up by lung epithelial cells. This work demonstrates a non-invasive, safe and effective approach to alleviate pulmonary fibrosis, but adverse effects such as cytotoxicity of uncertain mechanisms have emerged. But in general, the nanostructured lipid carrier has good biocompatibility [54], low toxicity [55], good inhalability [56], and slow-release control properties [57].

## Nanovesicles

**Fig. 10:**
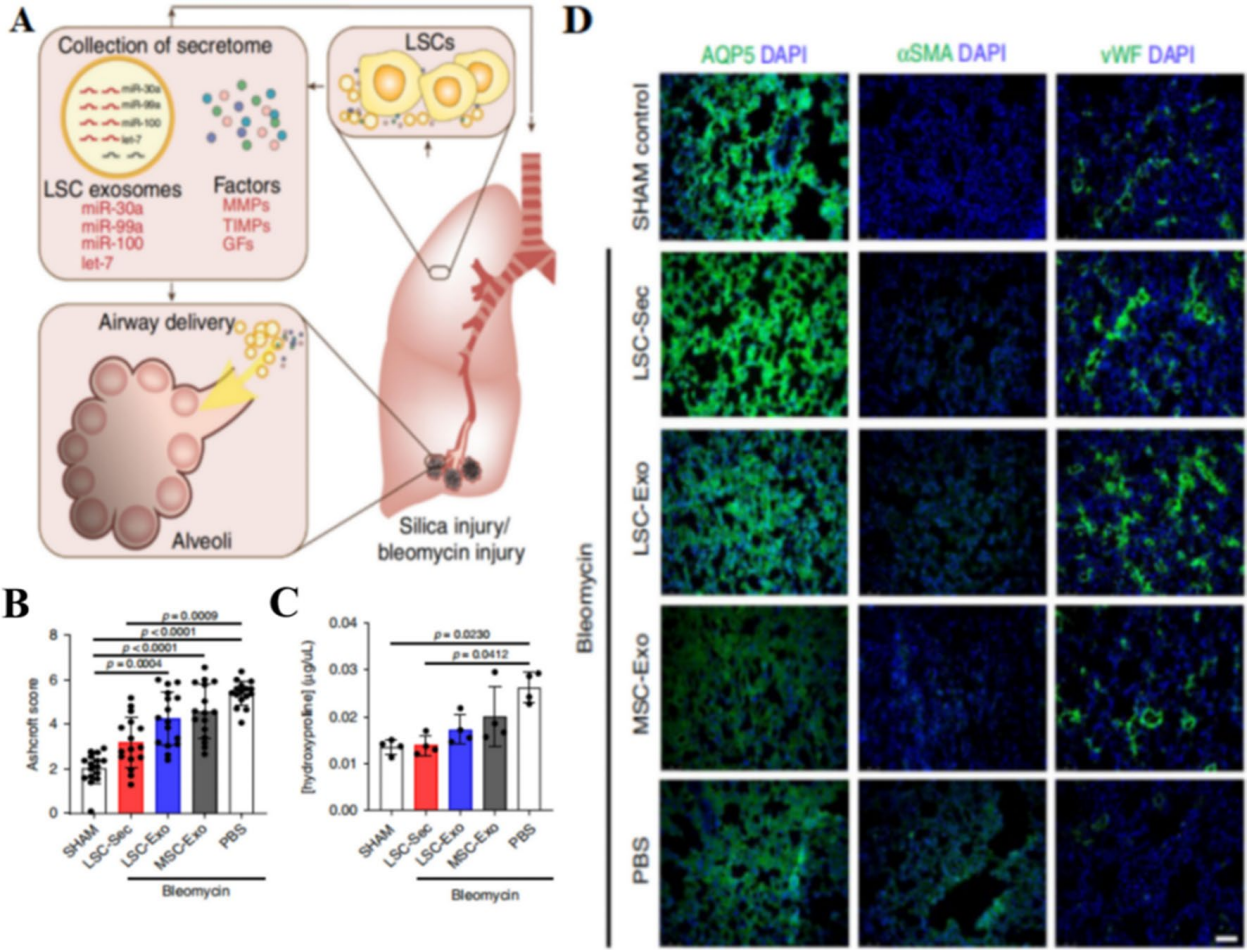
**A** Principal component analysis plots of LSC-exosome and MSC-exosome microRNA contents. **B** Quantification of fibrosis by Ashcroft score. The LSC-Exo treatment group showed therapeutic effects both in terms of maintaining normal lung structure and reducing fibrosis. **C** Quantification of pulmonary HYP levels; The LSC-Exo treatment group showed therapeutic effects in both reducing HYP levels and reducing collagen deposition. **D** LSC-Exo treatment group attenuated alveolar epithelial and vascular damage and reduced fibrosis, indicating an increase in AQP5+ and vWF+ cells and a decrease in αSMA+ cells [59]

Nanovesicles have lately attracted a lot of attention due to their potential as a medicine delivery method [58]. They have distinct benefits as naturally occurring endogenous drug carriers, including low immunogenicity, high blood stability, high delivery efficiency, targeting ability, and improved permeability and retention impact. Dinh et al. [59] put forward using lung spheroid cell exosomes (LSC-Exo) inhalation to treat two models of PF induced by bleomycin and silica, respectively. By restoring normal alveolar structure, lowering collagen buildup and myofibroblast proliferation, enhancing AQP5+ and vWF+ cells, and decreasing αSMA+ cells, LSC-Exo treatment eased and reversed bleomycin- and silica-induced fibrosis. Inspiratory capacity and respiratory compliance are improved significantly after the LSC-Exo remedy. In addition, the therapeutic effect of LSC-Exo were superior to other components derived from mesenchymal stem cells (MSC), confirming the therapeutic potential of LSC-Exo inhalation for lung regeneration (Fig. 10). Li et al. [60] discovered that inhalation of four doses of the human lung spheroid cells-nanodecoy can effectively alleviate the inflammatory cells infiltration, reduce the degree of PF caused by COVID-19. This nanodecoy is highly translatable, so progenitor cells serve as a potential therapeutic approach for the treatment of PF. Additionally, Ashcroft score shows that this LSC-nanodecoy largely reduces fibrosis in primate cynomolgus macaques.

## Polymeric nanocarriers

Polymeric nanocarriers can be divided into polymeric nanoparticles, micelles, dendrimers, and polymeric drug complexes [61]. Polymeric nanoparticles are now being studied intensively for their exceptional potential as an anti-fibrosis medication delivery strategy. They’re made by encapsulating, dissolving, and entrapping the medication in biodegradable polymers or embedding the molecule in a polymeric matrix.

Chitosan nanoparticles are nowadays of great interest in nanomedicine and in the development of novel therapeutic drug delivery systems with higher bioavailability, by improving specificity and sensitivity and reducing pharmacological toxicity [62]. Chitosan nanoparticles combine the natural properties of polymers with the possibility of tunable size and surface modification according to tailored needs [63]. According to Rashidipour et al. [64] paraquat herbicide loaded on Pectin/Chitosan/Tripolyphosphate nanoparticles effectively reduced acute lung damage and PF, implying decreased apoptosis, oxidative stress, and α-smooth muscle actin (α-SMA) expression in lung tissue. The nanoparticles themselves displayed little or no harm, as evidenced by inflammatory and apoptotic indicators and histology scores. Zhang et al. [65] used amphoteric phosphorylcholine and chitosan to develop mimetic phosphorylcholine chitosan nanoparticles (PCCs-NPs) as a protein (msFGFR2c) delivery platform for the treatment of PF, which inhibited TGF-β1-induced α-SMA expression in fibroblasts, significantly reduced PF scores and collagen deposition, and significantly improved survival rates.

**Fig. 11:**
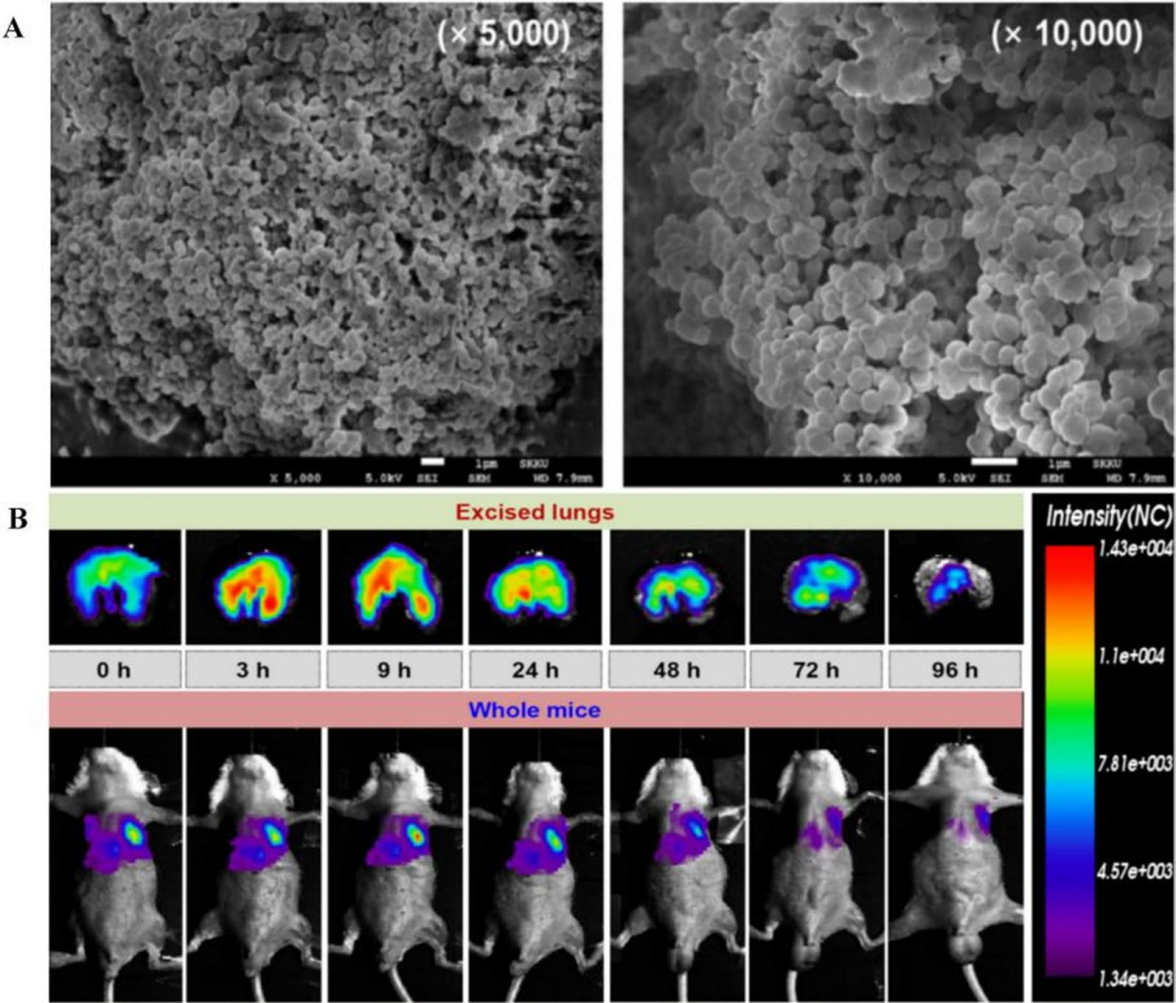
Effectiveness of PF treatment after inhalation of TAC PLGA-NPs. **A** Morphologies of chitosan TAC PLGA-NPs by field-emission scanning electron microscopy (FE-SEM). The mean size of TAC PLGA-NPs and chitosan TAC PLGA-NPs was 320 ± 9.0 and 441 ± 11.9 nm. **B** This nanomedicine demonstrated good localization and deposition rates in the lungs. This system is also perceived as an efficient sustained-release type inhalation system [67]

Poly(lactic-co-glycolic acid) is an FDA approved polymeric material with biodegradable and biocompatible characteristics [66]. Lee et al. [67] found that using an emulsification diffusion method prepared inhaled tacrolimus-loaded chitosan-coated-PLGA nanoparticles markedly reduced inflammation. The results showed tacrolimus extended release up to 5 days and demonstrated good localization and deposition rates in the lungs. This system is also perceived as an efficient sustained-release type inhalation system (Fig. 11). Elkomy et al. [68] developed a novel, non-invasive inhalable nifedipine-loaded chitosan-PLGA polymeric nanoparticles with the 61.81% entrapment efficiency and 50.4% sustained release profile over 24 h, which can regulate the TGF-β/β-catenin pathway in the rat model of bleomycin-induced PF.

Pirfenidone is the first licensed PF therapy, commonly used orally, and is known to mediate its anti-inflammatory and anti-fibrotic effects through modulation of cytokines and growth factors, but adverse effects such as gastrointestinal and photosensitivity are common in clinical trials, and dose limiting treatment leads to reduced effectiveness of the drug [69]. Therefore, the combination of pirfenidone and nanoparticles can be considered for inhalation administration to reduce side effects. Trivedi et al. [70] employed lactide-glycolide nanoparticles containing pirfenidone to ameliorate bleomycin-induced PF mice by intratracheal administration. The results indicated that the number of fibrotic cells, lymphocytes and neutrophils markedly decreased. These polymeric nanocarriers maintained delivery of pulmonary pirfenidone and enhance its antifibrotic efficacy.

The important role of the stromal cell-derived factor-1/chemokine receptor CXCR4 (SDF-1/CXCR4) axis in fibroblast recruitment and other pro-inflammatory and pro-fibrotic activities has been reported, and CXCR4 inhibition is a promising therapeutic target for PF [71–73]. Ding et al. [74] reported the development of a multimeric complex based on CXCR4 inhibition of poly(ethylimine) derivatives (PEI-C) for lung delivery of siRNA to silence fibrinogen activator inhibitor-1 (siPAI-1) in a combination therapy for PF, with fluorescence showing the longest drug retention time in the lung, significant downregulation of PAI-1 expression, and significant reduction in intrapulmonary collagen deposition.

## Protein nanocarrier

Protein nanocarriers use proteins as carriers to deliver drugs by reacting with the amino carboxyl groups on the surface of the protein. It can improve the stability, bioavailability and pharmacokinetics of therapeutic proteins. Seo et al. [75] investigated a sustained release type of inhalable albumin nanoparticles binding tacrolimus with 85.3 ± 4.7% encapsulation efficiency, direct exposure, rapid absorption, long deposition. The system significantly decreased the extent of fibrotic and appeared similar to untreated lungs.

**Fig. 12:**
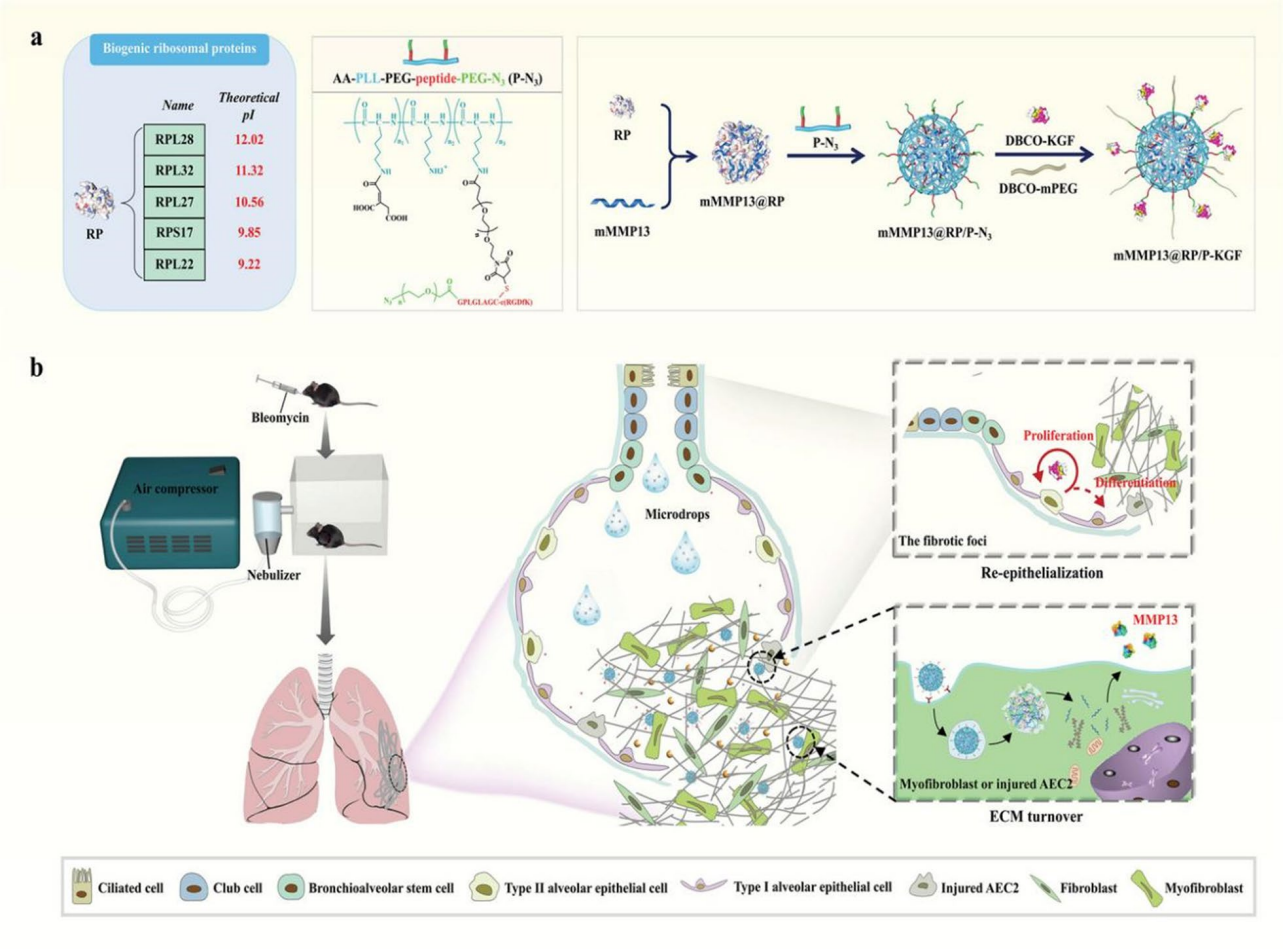
**A** Feasibility of recombinant endogenous ribosome proteins being screened for mRNA delivery; A nano-based delivery system containing mMMP13 and keratinocyte growth factor (KGF) was prepared by dual functional modification; **B** The nano drug deposited in alveoli after inhalation releases outer KGF in response to the lesion site and promotes the proliferation of alveolar epithelial cells. After uptake of the recombinant ribosomal protein complex of mMMP13, in situ production of MMP13 accelerates the degradation of extracellular matrix in the alveolar cavity and promotes fibrosis lesion repair [76]

Zhang et al. [76] constructed an mRNA nano-delivery system co-loaded with matrix metalloproteinase 13 mRNA (mMMP13) and keratinocyte growth factor through dual functionalization modification. The system can be used to repair fibrotic lesions by synergistically promoting matrix degradation and alveolar epithelial reconstruction in PF lesions, providing a new idea for the treatment of PF diseases (Fig. 12). Bai et al. [22] developed a mucus-permeable nanoparticle system that combines siRNA against IL-11 and is regulated by inhibition of extracellular signal-regulated kinase and SMAD2. Thereby inhibiting fibroblastic differentiation and reducing excessive extracellular matrix deposition, significantly reducing fibrosis development.

## Nanosuspension

**Fig. 13:**
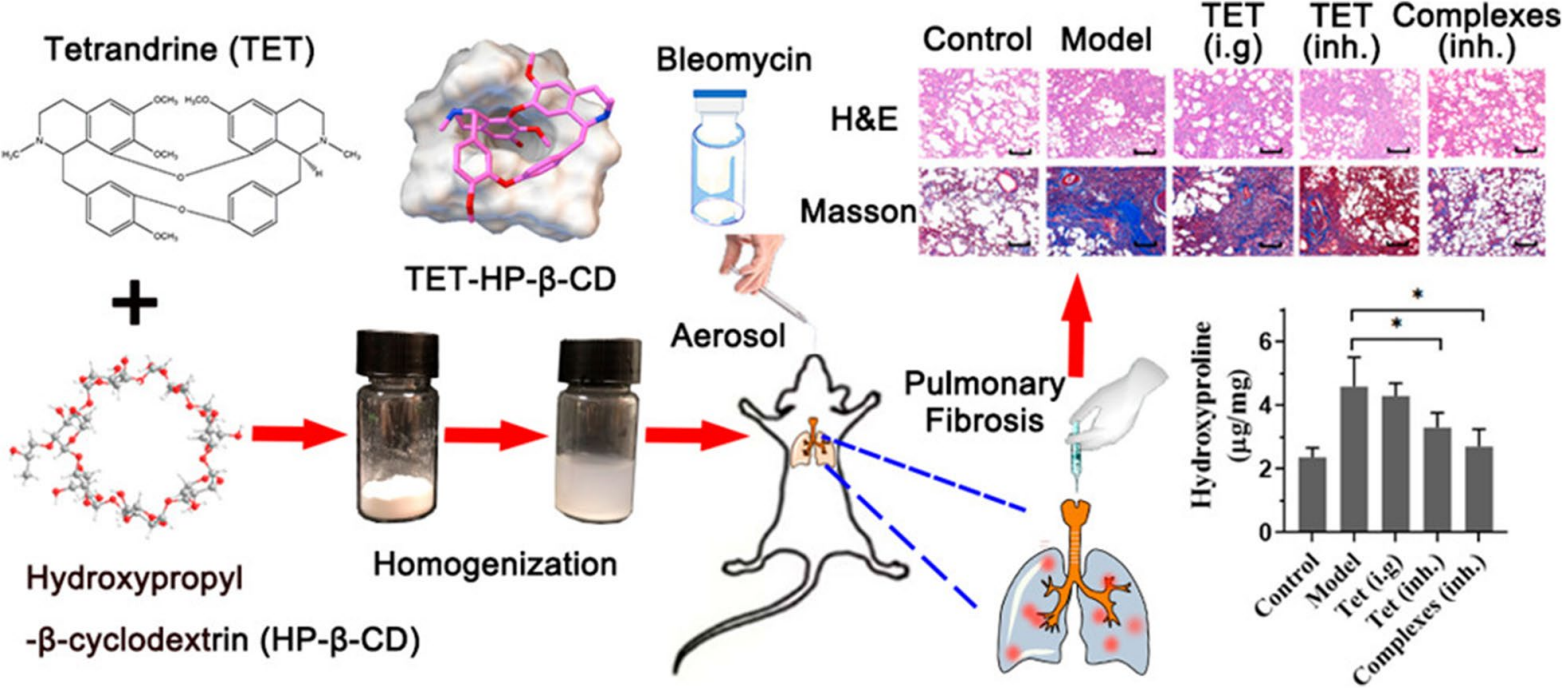
Schematic diagram of an inhalable powdered Tetrandrine-hydroxypropyl-β-cyclodextrin inclusion body nanosuspension for bleomycin-induced PF treatment [78]

Nanosuspension drug delivery system refers to the medicinal dispersion system formed by suspending nano drugs in solvents. It has evolved in response to the numerous water-insoluble drug candidates that have emerged through high-throughput drug screening procedures that place a premium on fit into hydrophobic receptor pockets [77]. Su et al. [78] utilized high-pressure homogenization method and developed an inhalable tetrandrine-hydroxypropyl-β-cyclodextrin inclusion nanosuspension (640.3 ± 49.2 nm) for bleomycin-induced PF therapy, indicating faster uptake, higher solubilizing efficiency, reducing inflammation and fibrosis level, limiting the accumulation of HYP in the lungs, modulating proteins expressed in the development of fibrosis, and better postoperative survival (Fig. 13).

## Nanoparticles

**Fig. 14:**
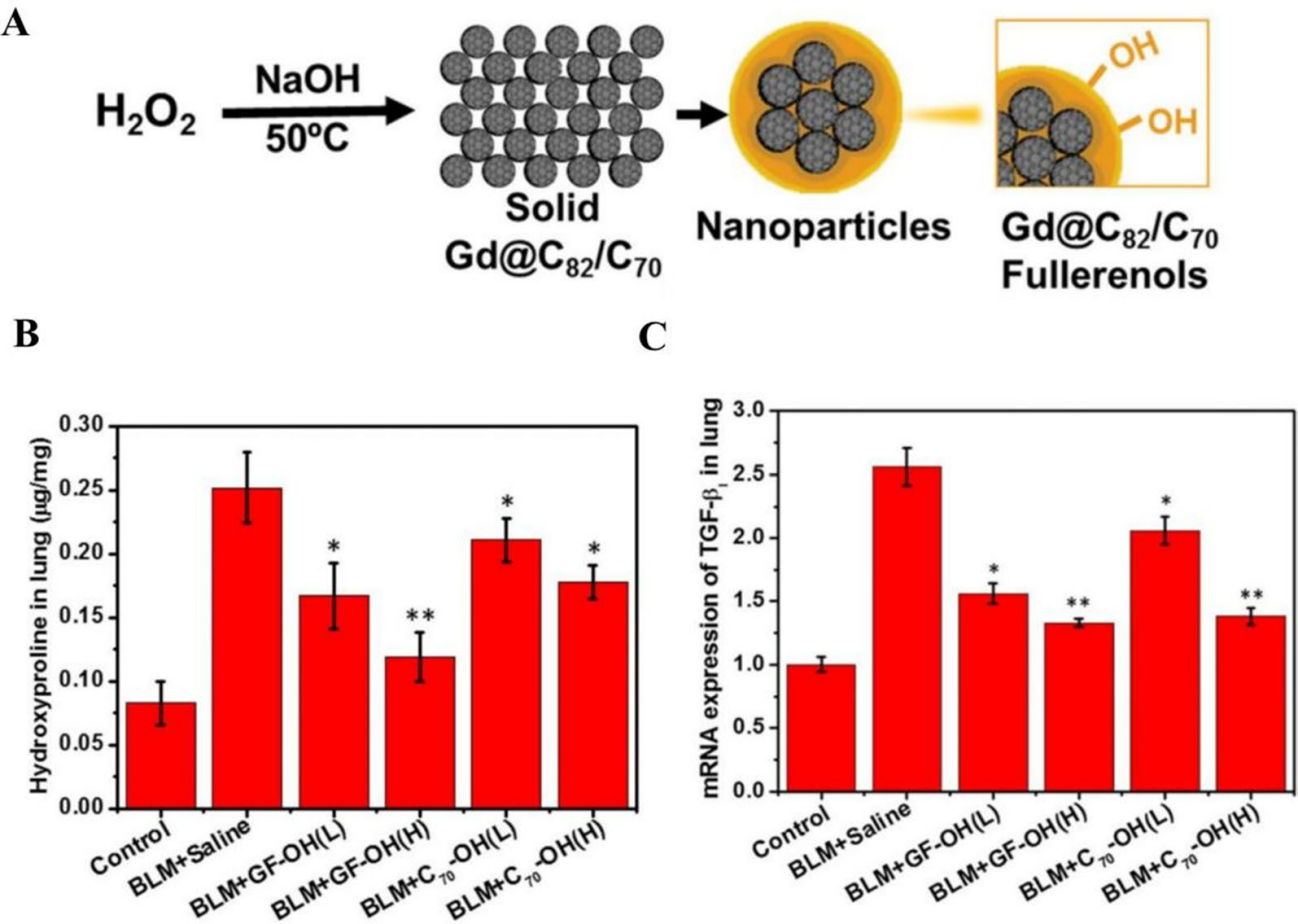
**A** Schematic diagrams of the synthesis of gadofullerenol and [70] fullerenol nanoparticles. **B** Comparison of HYP content in lung tissue. **C** Comparison of mRNA expression of TGF-β1 in lung [83]

Nanoparticles offer new possibilities for improving drug release properties and obtaining optimal results in the treatment of inflammatory lung diseases by modifying the physical properties of the inhaled drug delivery carriers, such as particle size, shape, density, surface charge and other surface modifications [79, 80]. Vartiainen et al. [81] fabricated a dry powder nanoparticle formulation that contained 29.4% of tilorone, 9.6% of leucine and 61.0% of mannitol for silica-induced PF treatment. The formulation promoted more stable penetration through the monolayer of lung cells. Additionally, it is important to note that there have been no studies on the pharmacokinetics of tirolone lung administration. Hemmati et al. [82] synthesized nano-curcumin with cyclodextrin as a neutral soluble carrier (275 nm), and the system can reduce the overall HYP content, TNF-α, TGF-β, platelet derived growth factor (PDGF) levels and increased IL-10 level. Compared to oral and conventional inhaled curcumin, nano-curcumin is more effective and has fewer side effects, significantly lower levels of inflammatory markers, and most importantly, inhalation of synthetic nano-curcumin can restore lung HYP levels. Zhou et al. [83] prepared inhalable gadofullerenol and fullerenol by a one-pot reaction, which obviously reduced collagen deposition caused by acute lung injury. This study revealed these nanoparticles’ antioxidant and anti-inflammatory function in regulating ROS-mediated inflammatory processes. Its therapeutic effects may be related to a synergistic mechanism of free radical scavenging and indirect regulation of TGF-β1 expression (Fig. 14).

## Gold nanoparticles

**Fig. 15:**
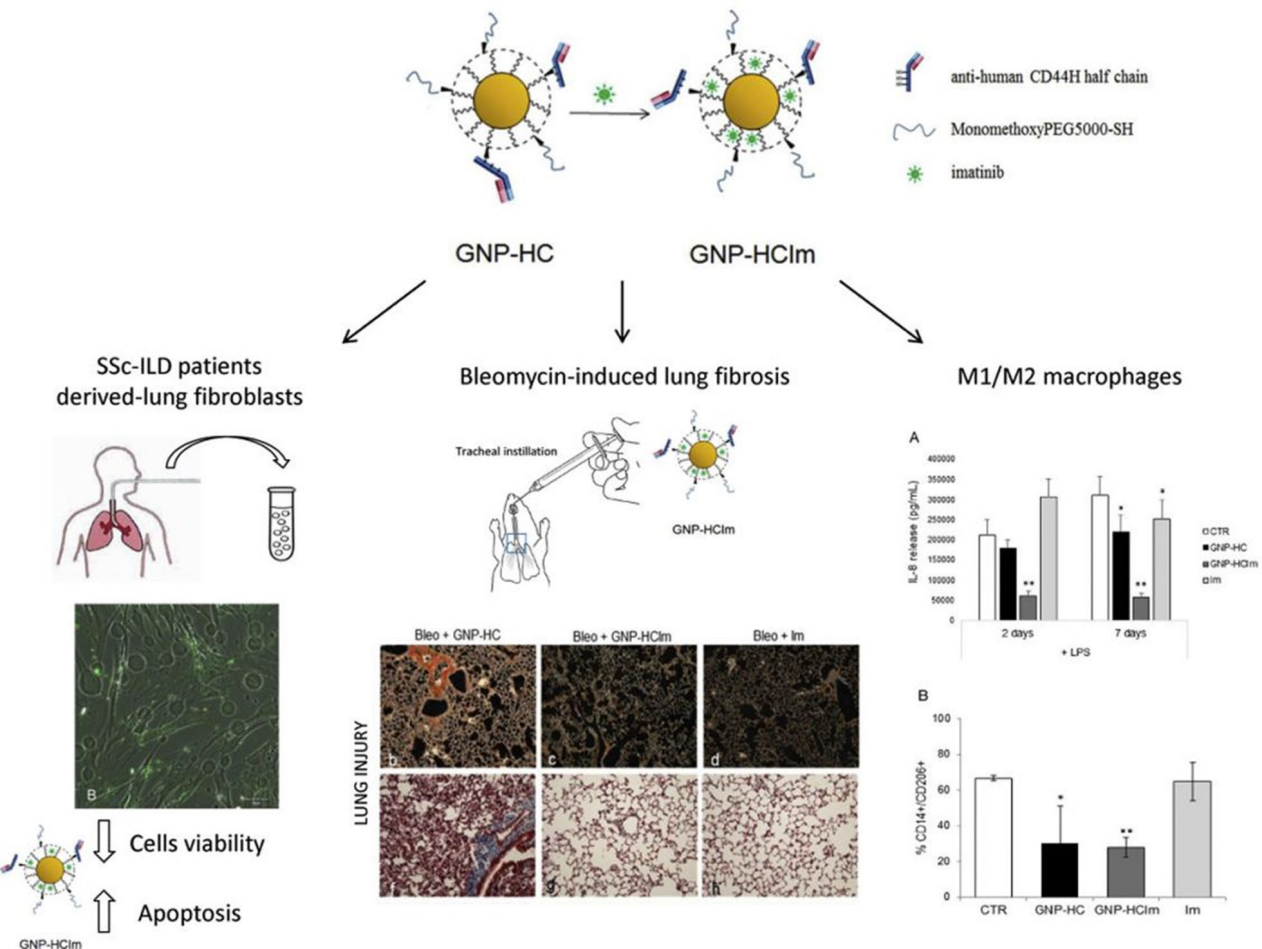
Schematic diagram of imatinib-loaded gold nanoparticles inhibiting the proliferation of fibroblasts and macrophages and ameliorating experimental bleomycin-induced PF in patients with systemic sclerosis [85]

Nanoscale gold surfaces have special effects. Therefore, gold nanoparticles can directly or indirectly produce different biological activities [84]. Codullo et al. [85] synthesized anti-CD44 imatinib-loaded gold nanoparticles, which acted on inhibition of proliferation and activity of pulmonary fibrotic cells, available reduction of IL-8 release, viability and polarization of M2 in alveolar macrophages (Fig. 15). Pandolfi et al. [86] have reported inhalable imatinib loading gold nanoparticles, which functionalized with antibody against CD44, demonstrating significant reduction tracheal lumen obliteration and apoptosis, and TGF-β-positive signal in surroundings.

## Hydrogel-based delivery

Due to the high biocompatibility [87], drug protection [88], spatial and temporal control of drug release [88] and physicochemical customisability [89] offered by hydrogels hydrogels are increasingly investigated as topical drug delivery systems. Shamskhou et al. [90] innovated a hydrogel-based delivery system for recombinant interleukin-10 (IL-10) that breaks the half-life limit of free IL-10, which has the ability to reversibly bind IL-10 through the use of heparin without bleeding or other complications. This formulation is remarkably more effective than free IL-10 for both preventing and reducing collagen deposition in the lung parenchyma after 7 days of inhalation and has been shown to be used to prevent and reverse bleomycin-induced PF (Table 2).

**Table 2:** Inhalation nano-drug delivery systems in PF therapy

Nano type	Drug carrier	Encapsulated Drug/DNA/RNA	Treatment effect	Refs
Liposome	Egg phosphatidylcholine, cholesterol	PGE2	Reduced the extent and severity of the fibrotic process	[40]
Liposome	/	Dexamethasone	Help to anti-COVID-19 complications, anti-oedema activity	[41]
Liposome	l-Arginine and lecithin	Salvianolic acids	AUC_(0–1)_was 2099.12 times larger than intravenous injection	[43]
Liposome	Mannitol	Budesonide, colchicine	Large numbers of inflammatory cells, alveolar septal thickness, fibrosis scores were significantly reduced	[44]
Liposome	Phosphatidylcholine	Naringin	Naringin liposomes maintained airway patency during administration and were effectively deposited in the lungs in 79% of the aerosol deposition assays	[45]
Liposome	1,2-Dipalmitoyl-*sn*-glycero-3-phosphocholine, cholesterol	Hyaluronic acid	Decreased IL-1β, IL-12, and VEGF transcripts	[47]
Liposome	Cholesterol, distearoyl phosphatidylcholine, 1,2-dimyristoyl-rac-glycero-3-methoxypolyethylene glycol-2000	MBD2 siRNA	Over 90% entrapment efficiency, no toxicity	[48]
Liposome	Polyethylene glycol	\	Compared to blank liposomes, PEGylated liposomes showed a slower decrease in fluorescence intensity, higher accumulation of bronchoalveolar lavage fluid and lower accumulation of alveolar macrophages such as RAW264.7 cells	[50]
Nano-structured lipid carrier	Precirol ATO5, squalene, SPC, DOTAP	PGE2, siRNA	Restricted lung fibrotic tissue damage, downregulated CTGF, TGFB1, TGFB2, TGFB3, TGFBR1, TGFBR2	[52]
Nano-structured lipid carrier	1,2-dimyristoyl-sn-glycero-3-phosphocholine (DMPC) and 1,2-dihexanoyl-sn-glycero-3-phosphocholine (DHPC)	STAT3 phosphorylated cell permeable peptide inhibitor (APTstat3-9R)	Inhibits differentiation of lung epithelial cells and fibroblasts to myofibroblasts and M2 polarization of macrophages, penetrates the lung surfactant barrier and is taken up by lung epithelial cells	[53]
Exosome	Human lung spheroid cell	miRNA	Protected apoptosis induced PF, demonstrated potential to treat lung regeneration in experimental models	[59]
Exosome	Human lung spheroid cell	\	Promotes viral clearance and reduces lung damage, and may be a potential drug for the treatment of COVID-19	[60]
Polymeric nanocarriers	Pectin, chitosan, tripolyphosphate	\	Reduced acute lung damage and PF, implying decreased apoptosis, oxidative stress, and α-SMA expression	[64]
Polymeric nanocarriers	Biomimetic phosphorylcholine-chitosan	msFGFR2c	Inhibition of TGF-β1-induced α-SMA expression significantly reduced lung fibrosis scores and collagen deposition, significantly improving survival	[65]
Polymeric nanocarriers	Chitosan-PLGA	Tacrolimus	Markedly reduced inflammation, demonstrated good localization, slow-release and deposition rates in the lungs	[67]
Polymeric nanocarriers	Chitosan-PLGA	Nifedipine	Regulated the TGF-β/β-catenin pathway	[68]
Polymeric nanocarriers	Lactide-glycolide	Pirfenidone	Decreased the number of fibrotic cells, lymphocytes and neutrophils markedly, and enhance pirfenidone antifibrotic efficacy	[70]
Polymeric nanocarriers	CXCR4-inhibiting poly(ethylenimine) derivative (PEI-C)	Silence plasminogen activator inhibitor-1 (siPAI-1)	Significantly downregulate PAI-1 expression and a significantly reduce collagen deposition in the lung, prolonged retention	[74]
Protein nanocarriers	Albumin	Tacrolimus	Improved lung inflammation and decreased fibrosis	[75]
Protein nanocarriers	Ribosomal protein-condensed mRNA core	mMMP13, KGF	Reduces the level of lung tissue fibrosis and synergistically restores alveolar integrity	[76]
Protein nanocarriers	PLGA-PEG	siRNA	Inhibition of ERK and SMAD2 inhibits fibroblast differentiation and reduces ECM deposition	[22]
Nano suspension	Cyclodextrin	Tetrandrine	High pulmonary concentrations, direct exposure, rapid absorption, long deposition	[78]
Nanoparticle	Leucine, mannitol	Tilorone	Promoted more stable penetration through the monolayer of lung cells	[81]
Nanoparticle	Cyclodextrin	Curcumin	Reduced TNF-α, TGF-β and PDGF level	[82]
Nanoparticle	Hydrogen peroxide, sodium hydroxide	Gadofullerenol, [70] fullerenol	Showed the antioxidant and anti-inflammatory functions	[83]
Gold nanoparticle	Antibody against CD44	Imatinib	Inhibit proliferation and activity of pulmonary fibrotic cells, available reduce IL-8 release, viability and polarize M2 in alveolar macrophages	[85]
Gold nanoparticle	Antibody against CD44	Imatinib	Reduced tracheal lumen obliteration and apoptosis	[86]
Hydrogels	Hyaluronan and heparin-based hydrogel	IL-10	Significantly outperformed soluble IL-10 in preventing and reducing collagen deposition in the lung parenchyma	[90]

## Conclusions and future outlook

PF is one of the most dangerous interstitial lung disorders with the poorest prognosis. It is becoming increasingly regarded as a complication in individuals who have been infected with COVID-19, highlighting the importance of finding effective and site-specific therapeutics to reverse and maybe cure the disease [91, 92]. At present, all drugs that can be used for the treatment of PF are administered by systemic administration, with large toxic and side effects and poor patient compliance. Effective inhaled drugs are emerged and developed. However, inhaled free anti-fibrosis agents are highly toxic, easily cleared quickly, and their distribution in the lungs is not specific.

Inhaled nano-based drug delivery systems have many advantages over conventional drug or micron delivery systems for the treatment of PF [93]: (1) Targeted drug delivery [94]: increases the concentration of drug in the lungs, controls the release of drug, overcomes the lung barrier, reduces the dose of drug thus reducing the side effects of treatment and improving patient compliance; (2) Increased bioavailability of drug [95, 96]: allows better water solubility and protects it from degradation; (3) Rapid onset of action [97]: avoids first pass effects in the liver. Nanovaccines can exploit the large surface area of the lung and the rich environmental characteristics of antigen-presenting cells to elicit robust immune defense against various mucosal diseases [98]. A small number of inhaled nanomedicines for lung diseases have entered clinical trials, such as LQ036 nanobodies for moderate to severe asthma, but they have been terminated in Phase I [99], and mRNA VX-522 for cystic fibrosis for cystic fibrosis is in Phase I clinical trials [100]. However, inhaled nanomaterials for PF have not yet started clinical trials and need further research.

But still, the development of inhaled nano-based drug delivery system presents many challenges. In addition to the safety and efficacy of inhaled drugs in the lung, the lung biopharmaceutical properties should also be fully evaluated in drug design to ensure that inhaled nano-based drugs can maintain pharmacodynamic concentration in lung tissue for a long time. Noteworthy, actively-targeted inhaled nano-based drug delivery system in the treatment of PF ensure that all of these are in the investigational or unattended stage. Additionally, nanotechnology as a novel approach has not yet been approved for the clinical treatment of PF. Further investigation can focus on nanomedicines, immunotherapy and inhalable nanovaccines, which hold great promise for the treatment of PF. In conclusion, the use of inhaled nano-based drug delivery system has great potential when it comes to PF therapy, the safety, efficacy and industrial production of its clinical application still need to be further studied.

## Data Availability

The dataset supporting this review article is included within all the cited articles.
